# Self-Perception of Physical Function Contributes to Participation in Cognitively- and Physically-Demanding Activities After Stroke

**DOI:** 10.3389/fneur.2020.00474

**Published:** 2020-06-09

**Authors:** Marjorie L. Nicholas, Kari Burch, Julianne R. Mitchell, Annie B. Fox, Carolyn M. Baum, Lisa Tabor Connor

**Affiliations:** ^1^Department of Communication Sciences & Disorders, MGH Institute of Health Professions, Boston, MA, United States; ^2^Program in Occupational Therapy and Department of Neurology, Washington University School of Medicine, St. Louis, MO, United States; ^3^Department of Occupational Therapy, MGH Institute of Health Professions, Boston, MA, United States; ^4^Center for Interprofessional Studies and Innovation, MGH Institute of Health Professions, Boston, MA, United States; ^5^Program in Occupational Therapy and Departments of Neurology & Social Work, Washington University in St. Louis, St. Louis, MO, United States

**Keywords:** stroke, activity participation, patient-reported outcome assessment, community reintegration, aphasia, cognition, physical function and mobility

## Abstract

**Background:** Persons with and without aphasia experience decreased participation in meaningful activities post-stroke that result in reduced autonomy and poorer quality of life. Physical, cognitive, and/or communication deficits are prevalent post-stroke and many activities given up are purported to require high levels of communicative, cognitive, or physical skill. However, the relationship between deficits after stroke and participation in life activities that appear to require high skill levels in these three areas has not been investigated fully.

**Objectives:** The objectives of this study are to: (1) determine differences in reported participation in communicatively-, cognitively-, or physically-demanding activities in persons after stroke with and without aphasia living in the community, and to (2) investigate whether performance on commonly used self-perception assessments of these three areas predicts reported participation in activities requiring higher levels of skill in these domains.

**Methods:** In a cross-sectional design, 82 individuals at least 6 months post-stroke with (*N* = 34) and without aphasia (*N* = 48) were administered a battery of neuropsychological and participation-based assessments. Supported communication techniques maximized inclusion of individuals with aphasia. A series of regression analyses investigated the relationship between self-perceived communicative, cognitive, and physical functioning and reported participation in activities post-stroke that required high amounts of skilled function in these areas.

**Results:** People with and without aphasia did not differ in terms of the percentage retained in communicatively-, cognitively-, or physically-demanding activities. All individuals retained higher levels of participation in communicatively- and cognitively-demanding activities (at least 60% retained), compared to participation inphysically-demanding activities (about 50% retained). The strongest predictor for retaining participation in two of the three domains of activities was self-perception of physical function, though much of the variance remained unexplained. Self-perception of communication was not related to participation retention in any of the three domains.

**Significance of Impact:** Rehabilitation professionals should be aware of the impact that a variety of communicative, cognitive, and physical factors may have on participation post-stroke. Self-perceptions of impairments in communication and cognition may not directly predict participation in activities requiring high levels of communicative and/or cognitive skill, at least for those with mild impairment, even though activities requiring those skills are given up or done less after stroke.

## Introduction

Stroke is the fifth leading cause of death in the US, with an annual prevalence of 795,000 (1). Aphasia, characterized by difficulty in producing and understanding spoken language, reading, and writing, occurs in 25–40% of stroke cases, and is estimated to currently affect ~2 million Americans (2). After stroke, both persons with aphasia (PWA) and without aphasia (PWOA) experience diminished participation in everyday life and in their meaningful daily activities and role functioning [e.g., Foley et al. (3)]. Understanding the factors that enhance or inhibit participation post-stroke is imperative to enable PWA and PWOA to live satisfying, meaningful lives. Although many persons who have had a stroke experience diminished participation in everyday life activities, the extent to which community-dwelling PWA are able to resume pre-stroke roles and participate in meaningful activities after stroke has received considerably less attention in the literature (4–8) than studies examining PWOA because PWA are largely excluded from research due to their communication deficits (9).

Participation is a complex construct defined broadly in the World Health Organizations's International Classification of Functioning and Disability as “involvement in a life situation,” arising from an interaction among body structures and body functions, environmental factors, personal factors, and activity demands (10, 11). After a stroke, participation has been measured by using assessments of retention of previous activities, reintegration in the home and community, and perceived recovery from the stroke (12–14). Participation restrictions post-stroke often result in reduced autonomy and poorer quality of life [e.g., Hartman-Maeir et al. (15)], consistent with an occupational perspective that participation in everyday activities is required to improve and maintain health and well-being (16, 17).

In addition to chronic physical impairments, persistent communicative and cognitive stroke sequelae affect more than 50% of the community-dwelling stroke population and result in diminished activity engagement; the majority of individuals report lacking even one important and meaningful activity to do each day (14, 15, 18). Even 6 months or more after mild stroke, individuals report decreased participation in meaningful activities, including work, volunteering, travel, and socialization (3, 19, 20).

PWA may be increasingly susceptible to reductions in participation, as activity engagement may be associated with aphasia severity (21–23). Several studies have identified that the majority of these individuals have difficulty with instrumental activities of daily living (IADL) after stroke (14, 15, 24, 25). Common examples of difficult post-stroke activities that are characterized as “complex” IADLs include household tasks such as meal preparation, housekeeping, laundry, driving, and socializing (14, 15). These activities vary with respect to how communicatively- and/or cognitively-demanding they are. Some activities like doing laundry may have fewer demands in these areas, while others like socializing with friends and family or conducting transactions such as shopping or banking, clearly have higher communicative and cognitive demands.

The ability to perform IADLs post-stroke, as well as return to community activities, is associated with improved life satisfaction as well as health-related quality of life (HRQOL), a construct encompassing physical, non-physical (communicative and cognitive), social, and role functioning as well as subjective experiences of health and well-being (14, 15, 26, 27). Further, participation is a significant predictor of life satisfaction after stroke, beyond that which is accounted for by variables such as depression (15). On the negative side, long-term dissatisfaction, and decreased quality of life are associated with decreased activity engagement and participation after stroke (15).

Many of the activities found to be difficult post-stroke and which exert a powerful influence on HRQOL are frequently described as “complex” or “higher order” IADL, implying that the activities require higher levels of communicative and/or cognitive functioning (14, 15, 28). Aphasia, as an impairment of language affecting communication, may be considered as one of many areas that falls under the larger umbrella of cognitive impairments. Moreover, PWA may have concomitant deficits in executive functions, memory, attention, and visuospatial functions. Impairments in these areas seen in PWA may be chronic and to some extent under-treated because physical impairment is often the main focus in intervention post-stroke (15, 29–34).

An additional challenge that needs to be addressed is measuring the construct of participation, particularly in PWA who have difficulty with either verbal expression or comprehension of language. The Activity Card Sort (ACS) (35) is one measure that is ideally suited for people with impaired communication because it does not require overt language expression and can be easily adapted to require minimal language comprehension. The ACS consists of 89 photographs of activities, grouped into four categories: Instrumental, High Demand Leisure, Low Demand Leisure, and Social Activities. The ACS includes activities that represent common and likely-to-be valued activities that encompass a range of life pursuits. Individuals group the photographs into piles to indicate whether they have ever done the activity prior and whether they continue to do it now, among other types of sorts. Clinicians and researchers can determine which activities have been given up or retained post-stroke and can calculate percentage retained of the activities in each of the four activity domains. Several published studies have used the ACS to assess the extent to which people with various health conditions have retained their participation in activities (19, 36–43).

To understand better the nature of the demands of activities themselves and how those demands influence participation, an unpublished study was completed in our laboratory using the ACS. Forty-three healthy adult raters judged the extent to which nine dimensions of activity were needed to be able to participate in each of the 89 activities. The nine dimensions that each activity was rated on were: physical exertion, a partner to do the activity with, mobility, expressive communication, language comprehension, cognitive skills, fine motor skills, financial resources, and need for transportation.

To further understand factors affecting post-stroke participation in meaningful life activities, the current study used a subset of these dimensional scores obtained for the ACS activities to examine the question: What is the relationship between self-perception ratings of communication, cognition, and physical functioning post-stroke to participation in PWA and PWOA? We hypothesize that self-perception ratings of communicative and cognitive impairments will predict retention scores for items rated as requiring high levels of skill in these areas. For example, people with self-perceptions of greater communication impairment may show less retention of activities that are high in communication and/or cognitive demands; and conversely people who perceive themselves as having relatively preserved communication and cognitive skills may show greater retention of cognitively and communicatively-demanding activities (21). Both groups of participants are expected to show a decrease in retention of activities that are physically demanding if they perceive themselves as physically limited by the sequelae of their stroke. Conversely, some activities may be deemed more important to quality of life than others by participants so that they return to them despite having difficulty with multiple dimensions required to perform the activity. Thus, although we expect our general hypothesis to be supported, it is also possible that nuances will emerge across activities where this is not the case.

There are several different types of measures of impairment post-stroke, including (1) measures known as “patient-reported” or self-perception measures, commonly using various scales, questionnaires, or survey approaches; and (2) objective measures such as those often administered by clinicians to assess behavior that can be compared to group norms. Both means of assessment are undoubtedly important to obtaining a full and well-rounded assessment of contructs of interest. In this paper we focus on measures of self-perception of impairments as they relate to participation; a subsequent study will address a similar question using only objective measures.

## Materials and Methods

### Study Population

Participants were 34 people with aphasia and 48 people without aphasia who received medical services for a stroke at Barnes Jewish Hospital in St. Louis, Missouri, who lived in the community, and consented to have their data included in the Washington University Cognitive Rehabilitation Research Group's Stroke Registry or who participated in the research study at the MGH Institute of Health Professions in Boston, Massachusetts. Both sites received approval from their respective Institutional Review Boards and informed consent procedures were followed. Inclusion criteria for this study included: (1) six months or greater post-stroke, (2) ability to withstand two hours of testing, and (3) ability to commute to testing site by car or taxicab. Exclusion criteria included: (1) history of multiple strokes, (2) traumatic brain injury, (3) seizure disorder, (4) pre-stroke disability as evaluated by modified Rankin Scale (score of ≥ 2), (5) pre-existing neurological condition that could interfere with evaluation (e.g., MS, dementia, PD, ALS), or (6) severe medical or psychiatric illness. Written consent was obtained at testing.

#### People With Aphasia (PWA)

Inclusion criteria included: presence of aphasia by National Institute of Health Stroke Scale (NIHSS) aphasia item with a score > 0 at the acute hospital stay (*N* = 28) or who had received diagnostic confirmation of aphasia within the past 6 months (*N* = 6, all recruited from MGH Institute of Health Professions); and the capacity to give reliable yes/no responses.

Participants were screened over the phone to confirm that they were eligible to participate in this study. The aim was to include all who could provide a reliable yes/no response. The screening involved reading a story consisting of 3 brief sentences two times. Comprehension questions requiring yes/no responses were read. Candidates could indicate their yes/no response by any means they chose (e.g., a tap for “yes”). Those who answered 3 of 4 questions qualified for the study. If there was any doubt about eligibility, PWA were invited to the study and further evaluation was done with a consent comprehension assessment conducted with supported communication techniques [as described in Tucker et al. (44)]. Only if the participant was then unable to indicate comprehension of key elements of consent was that individual excluded from participating in the study. Two people screened were excluded as participants and no data on these individuals were collected.

Once enrolled in the study, PWA received the short form of the Boston Diagnostic Aphasia Examination-III (45) to characterize their language impairment with the Language Competency Index (LCI). These scores are included in Table 1. LCI Expression scores ranged from 22.5 to 100; LCI Comprehension scores ranged from 20 to 100; LCI Total scores ranged from 28.75 to 100. Three PWA obtained LCI scores of 100, indicating no language impairment. None of these 3 individuals rated themselves as not experiencing a communication deficit on the Stroke Impact Scale.

**Table 1:** Participant characteristics with means (M) and standard deviations (SD) in parentheses.

	**All (*n* = 82)**	**PWA (*n* = 34)**	**PWOA (*n* = 48)**
	**M(SD)/*n*(%)**	**M(SD)/*n*(%)**	**M(SD)/*n*(%)**
Age, mean (SD)	60 (12)	62 (12)	59 (12)
**GENDER AND EDUCATION**			
Men	28 (34)	14 (41)	14 (29)
Women	54 (66)	20 (59)	34 (71)
Education, years mean (SD)	15 (3.4)	15 (4.1)	14 (2.7)
**ETHNICITY**			
Caucasian	36 (44)	19 (56)	17 (35)
African American	45 (55)	14 (41)	31 (65)
Other	1 (1)	1 (3)	0 (0)
**LANGUAGE COMPETENCY INDEX**			
Expression		74.8 (21.8)	
Comprehension		75.5 (21.0)	
Total		75.1 (20.3)	

*PWA, People with aphasia; PWOA, People without aphasia; PWA were longer post-onset than PWOA (p = 0.013); Ethnicity differed marginally across groups (p = 0.07)*.

#### People Without Aphasia (PWOA)

Participants with stroke, but no aphasia as determined by the NIHSS aphasia item (a score of 0) at the acute hospital stay were included. All PWA and PWOA were participants in a larger investigation.

Table 1 describes characteristics of the participants. For the purposes of this study, the absolute value of *p* < 0.05 was considered statistically significant. There were no statistically significant differences between groups on age or education. Gender did not differ by group, *X*^2^ (2) = 1.3, *p* = 0.26. There was a marginally statistically significant different distribution of race/ethnicity by group, *X*^2^ (2) = 5.3, *p* = 0.07, with more African American participants in the PWOA group than in the PWA group.

## Measures

Self-report measures depend on the respondent's ability to process language both receptively and expressively. Aphasia can be a significant barrier for participating in subjective aspects of stroke outcome research (46–48). This study employed general principles for supportive techniques that can be utilized with any assessment without compromising the assessment's psychometric properties for PWA (44). These principles have been derived from prior studies on communication support: written support can increase auditory comprehension (49–51); reading comprehension can be enhanced by changing font style, size, and letter and line spacing (52, 53). Three types of supported communication techniques were used in this study: test administration modifications, response format modifications, and a systematic hierarchy of examiner supports [see Tucker et al. (44) for details]. Only two potential participants were not able to be included after using these supports; their data were excluded from this study.

Each participant was assessed with the objective assessment, the *National Institutes of Health Stroke Scale*, to characterize stroke impairment and the *Stroke Impact Scale* to assess self-perception of abilities across eight domains.

The *National Institutes of Health Stroke Scale* [NIHSS; (54)] was administered by certified assessors to ascertain cognitive, sensory, and motor impairments resulting from a stroke. The 13-item test is based on a score ranging from zero to 42; lower scores indicate lower levels of neurological impairment. Reliability is good to excellent and validity is high (55).

### Self-Perception of Abilities and Stroke Impact

*The Stroke Impact Scale, version 2.0* [SIS: (56)] assesses self-perceived impairments, disabilities, and participation following a stroke. The maximum score is 100. The eight domains of the SIS include: Strength, Hand Function, ADL/IADL, Mobility, Communication, Emotion, Memory and Thinking, and Participation/Role Function (56). The three domains included in this investigation were as follows: (a) as a measure of self-perception of physical functioning we averaged the scores from the Strength (four questions), Hand Function (5 questions), and Mobility (ten questions) domains; (b) as a measure of self-perception of communication we used the score from the Communication domain (seven questions), and (c) as a measure of self-perception of cognition, we used the score from the Memory and Thinking domain (8 questions). These scales have high reliability, with alphas ranging from 0.83 to 0.90. Inter-class correlation coefficients ranged from 0.70 to 0.92. Validity was established by correlating SIS domain scores with other measures of that function (57). The items on the SIS that were used for these measures are in Appendix II.

### Participation

The *Activity Card Sort* (ACS), 2nd Edition, Recovering version, was used as the dependent measure to assess participation in instrumental, social, and high- and low-physical-demand leisure activities (35). Participants group and sort each pictured item into categories that indicate whether they continue to do each activity, have given it up, do the activity less, or have started the activity since their stroke. Percent retained is the number of current activities, which is the number of activities they continue to do (1 point each) + do less (0.5 points each) + started (1 point each), divided by the number of previous activities, which is the number of activities they continue to do (1 point each) + the number of activities they have given up (1 point each). This ratio is then multiplied by 100 to obtain the percent retained score. The ACS has high internal consistency (α ≥ 0.83 for the 4 domains) (58). Test-retest reliability is high, with intra-class correlations ranging from 0.71 (58) to 0.98 (59). ACS scores have content, construct, and predictive validity (35). The internal consistency and construct validity of the new scales is unknown.

In the unpublished study mentioned earlier, 43 healthy adult raters, primarily occupational therapy students, and other volunteers who worked in the medical school environment, judged the extent to which nine dimensions of activity were needed for a person to be able to participate in each of the 89 activities of the ACS. The nine dimensions that each activity was rated on were: physical exertion, a partner to do the activity with, mobility, expressive communication, language comprehension, cognitive skills, fine motor skills, financial resources, and need for transportation. Each activity received a rating from 0 to 3 (none = 0, some = 1, a fair amount = 2, a lot = 3) on each of the nine dimensions. Average ratings for each item for each dimension were then calculated across the raters. Scores were then regrouped into three categories based on average ratings: The activity required a little amount (0–0.99); a fair amount (1–1.99); or a lot (>2.0) of the activity demand for the nine dimensions. Individual ACS activities may receive ratings of “*A lot”* across few, several, or most of the nine dimensions, resulting in a complex mix of requirements for each activity. To illustrate further, Table 2 shows the scores for two activities, *bicycling* and *talking on the telephone* on the dimensions that we considered in this study (communication, cognition, and physical functioning).

**Table 2:** Scores on the physical exertion, communication, and cognitive skill dimensions for two different activities: bicycling and talking on the telephone.

**Rated dimensions**	**Activity: bicycling**	**Activity: talking on the telephone**
Physical exertion	2.9	0.3
Communication	0.4	2.7
Cognitive skill	1.2	1.5

This study used items (activities) from the ACS in a novel manner to derive three participation scores for communicatively-, cognitively-, and physically-demanding activities, areas that many people experience difficulties with after stroke. While the 89 activities within the ACS are categorized into the four separate domains mentioned earlier, participation in each activity requires differing demands that may influence an individual's ability to participate in any given activity. For the purposes of the current study, the ACS activities were regrouped into categories involving demands in three particular dimensions: communicative, cognitive, or physical exertion requirements. Scores were found to be high (≥2.0) on one or more of these three dimensions on 59 of the ACS activities, comprising 2/3 of the ACS items. Results of this regrouping produced: (a) *High-Communitive items*- 35 activities requiring a lot of communicative skill; for this we used scores from the communication comprehension dimension, which happened to also include all items that were high on expressive communication. About half of these (17 items) were high on only the communication dimension (not cognition or physical), and half were high on communication plus at least one other dimension; (b) *High-Cognitive items*- 27 activities required a lot of cognitive skill and 5 of these were high on only the cognitive dimension. Many high-cognitive activities were also high on the communication dimension (18 of 27); (c) *High-Physical items*- 23 activities required a lot of physical exertion and the majority (15 items) of these were high on only this dimension. The specific ACS activities belonging to each category are found in Appendix I. Ratings for each item for each of the three dimensions and the percent retained for each item for the entire sample are shown in the appendix as well.

## Data Analysis

Separate linear regression models were used to examine three dependent variables measuring participation using the ACS: the percent retained activities for (a) communicatively-demanding activities, (b) cognitively-demanding activities, and (c) physically-demanding activities. The predictor variables used in the regression analyses were the three self-perception scores derived from the SIS questions relevant to communicative function, cognitive function, and physical function. In addition, months post onset (MPO) and the total NIHSS score were included as covariates. The hypothesis was that the predictors should account for variability in percentage retained on the ACS only for those items with high scores in the dimensions that matched the outcome domain. For example, perceived level of communication impairment (quite relevant because we explicitly included people with aphasia in our sample) should predict the extent to which people retain activities that are highly demanding of communication skills. Likewise, perceived cognitive abilities should only uniquely predict the extent to which individuals retain cognitively demanding activities and perceived physical function should uniquely predict the retention of physically demanding activities. Before testing the linear regression models, we compared PWA and PWOA on the dependent variables.

## Results

Scores obtained on each of the measures for all participants and separated into PWA and PWOA are displayed in Table 3.

**Table 3:** Means (M) and standard deviations (SD) for PWA and PWOA for self-perceptions, months post-onset, stroke severity, and participation percent retained.

**Measure**	**All (*N* = 82)**	**PWA (*N* = 34)**	**PWOA (*N* = 48)**
	**M (SD)**	**M (SD)**	**M (SD)**
**SELF-PERCEPTIONS AND OTHER PREDICTORS**			
SIS Physical Function	63.0 (25.5)	66.3 (27.6)	60.8 (24.1)
SIS Cognition	75.2 (21.9)	71.0 (21.4)	78.1 (22.0)
SIS Communication^*^	80.6 (21.0)	70.2 (22.1)	87.7 (17.2)
NIHSS Total Score	2.5 (2.2)	2.5 (2.4)	2.5 (2.1)
MPO^*^	23.6 (32.2)	34.5 (48.5)	16.4 (7.8)
**ACS ACTIVITIES PERCENT RETAINED**			
High Communicative Skill	74.9 (17.3)	75.2 (18.3)	74.7 (16.8)
High Cognitive Skill	64.5 (21.6)	67.2 (21.6)	62.6 (21.6)
High Physical Skill	47.2 (28.7)	50.4 (28.6)	44.9 (28.8)

* *Significant differences between groups: PWA, People with aphasia; PWOA, People without aphasia; SIS, Stroke Impact Scale, version 2.0; NIHSS, National Institutes of Health Stroke Scale; MPO, months post-onset; ACS, Activity Card Sort, version 2*.

### Differences Between Participants on the Dependent and Predictor Measures

Appendix I shows the percent-retained data post-stroke for each of the 59 activities that were considered in this investigation. Retention rates for the various ACS activities ranged from a low of 24% on item 65 “Playing tennis or racquet sports” and 26% on item 20 “Work (paid),” to a high of 98% on item 52, “Watching television.”

There were no statistically significant differences between groups (PWA, PWOA) on any of the three percent-retained participation scores (see Table 3), all *p*_s_ > 0.15: (1) *High-Communication items*, or (2) *High-Cognitive items*, or (3) *High-Physical items*. In terms of percent retained activities, most people post-stroke continued to participate in communicatively-and cognitively-demanding activities at moderately high rates, regardless of whether they had aphasia or not. Percent retained participation in physically demanding activities was somewhat less than 50% for both groups. Because there were no differences between the groups on the participation measures, regression analyses were conducted on the combined sample.

For measures that were included as predictors of participation on the ACS (see Table 3), only SIS Communication scores, *t*_(79)_ = 4.0, *p* = 0.001 and months post onset, *t*_(78)_ = 2.55, *p* = 0.013, differed by group with PWA reporting significantly lower self-perceptions of communication ability and being longer post-stroke onset. NIHSS scores did not differ between groups.

### Differences in Percent Retained Between ACS Scales

Because there were no group differences in ACS percent retained, we conducted analyses of the differences between ACS scales collapsing across group. Percent retained for ACS High Communicative Skill was greater than percent retained for ACS High Cognitive Skill, *t*_(31)_ = 9.19, *p* < 0.0001. Percent retained for High Cognitive Skill was greater than percent retained for High Physical Skill, *t*_(81)_ = 9.03, *p* < 0.0001. Percent retained for High Communicative Skill was greater than for High Physical Skill, *t*_(81)_ = 12.12, *p* < 0.0001.

### Relation of Self-Perception of Impairment in Three Domains to Participation

We examined whether self-perceptions of communicative, cognitive, and physical impairment predicted participation in these domains using linear regression models. For each outcome, we tested models with and without the inclusion of covariates (age, gender, education, and the NIH Stroke Scale Total score).

For participation in high-communicative activities, self-perception of *physical function* was significantly and positively related to participation, *ps* < 0.05, adjusted *R*^2^ = 0.27. Once covariates were included, however, the effect was no longer significant, indicating that no single variable predicted participation in high-communicative activities, adjusted *R*^2^ = 0.25. Results of the models are presented in Table 4.

**Table 4:** Regression models examining self-perceptions and participation in high-communicative activities with parameter estimates and standard errors in parentheses.

	**Communication tasks % retained**	
	**Without covariates**	**With covariates**
Intercept	41.504^***^	42.468^**^
	(6.216)	(14.991)
Age		−0.196
		(0.149)
Education		1.173
		(0.627)
Gender		−1.249
		(3.676)
NIH stroke scale		−0.693
		(0.940)
SIS physical	0.191^*^	0.153
	(0.074)	(0.084)
SIS memory	0.123	0.063
	(0.099)	(0.107)
SIS communication	0.148	0.194
	(0.101)	(0.108)
Observations	81	78
*R*^2^	0.298	0.314
Adjusted *R*^2^	0.271	0.245
Residual Std. Error	15.339 (df = 77)	15.270 (df = 70)
F statistic	10.916^***^ (df = 3; 77)	4.570^***^ (df = 7; 70)

* p < 0.05;

** p < 0.01;

*** *p < 0.001*.

For participation in *High-Cognitive activities*, self-perceptions of both *physical* and *cognitive function* were significantly and positively related to participation, *p* < 0.05, adjusted *R*^2^ = 0.34. Once covariates were included, only perception of *physical function* significantly predicted participation in *High-Cognitive activities, p* < 0.05, adjusted *R*^2^ = 0.33. Results of the models are presented in Table 5.

**Table 5:** Regression models examining self-perceptions and participation in high-cognitive activities with parameter estimates and standard errors in parentheses.

	**Cognitive skill % retained**	
	**Without covariates**	**With covariates**
Intercept	24.652^**^	32.349
	(7.425)	(17.541)
Age		−0.224
		(0.174)
Education		1.382
		(0.734)
Gender		1.390
		(4.302)
NIH stroke scale		−1.897
		(1.100)
SIS physical	0.348^***^	0.259^*^
	(0.089)	(0.098)
SIS cognition	0.307^*^	0.234
	(0.119)	(0.125)
SIS communication	−0.066	−0.050
	(0.121)	(0.127)
Observations	81	78
*R*^2^	0.365	0.394
Adjusted *R*^2^	0.340	0.334
Residual Std. Error	18.322 (df = 77)	17.867 (df = 70)
F statistic	14.739^***^ (df = 3; 77)	6.514^***^ (df = 7; 70)

* p < 0.05;

** p < 0.01;

*** *p < 0.001*.

For participation in *High-Physical activities*, self-perceptions of both *physical* and *cognitive function* were significantly and positively related to participation, *p*_s_ < 0.05, adjusted *R*^2^ = 0.22. Once covariates were included, *age* and self-perception of *physical function* were the only significant predictors of participation in physically-demanding activities, *p* < 0.01, adjusted *R*^2^ = 0.28. Results of the models are presented in Table 6.

**Table 6:** Regression models examining self-perceptions and participation in high-physical activities with parameter estimates and standard errors in parentheses.

	**Physical exertion skill % retained**	
	**Without covariates**	**With covariates**
Intercept	14.179	43.867
	(10.400)	(24.141)
Age		−0.690^**^
		(0.239)
Education		1.123
		(1.010)
Gender		1.964
		(5.921)
NIH stroke scale		−1.399
		(1.514)
SIS physical	0.420^**^	0.377^**^
	(0.124)	(0.136)
SIS cognition	0.391^*^	0.329
	(0.166)	(0.172)
SIS communication	−0.280	−0.210
	(0.170)	(0.174)
Observations	81	78
*R*^2^	0.247	0.347
Adjusted *R*^2^	0.218	0.282
Residual Std. Error	25.663 (df = 77)	24.590 (df = 70)
F statistic	8.439^***^ (df = 3; 77)	5.311^***^ (df = 7; 70)

* p < 0.05;

** p < 0.01;

*** *p < 0.001*.

## Discussion

Participants in this sample could be considered as having relatively mild strokes, based on their chronic NIHSS scores that ranged from 0 to 10 with a mean of 2.5. It is interesting and somewhat surprising, therefore, to note that their activity participation retention rates indicate they are giving up between 30 and 50% of their activities in the three groupings of activities examined in this study, that is, activities requiring high levels of communicative skill, cognitive skill, and/or physical exertion. However, contrary to our expectations, there were no statistically significant differences between groups based on presence/absence of aphasia (PWA, PWOA) on any of the three percent-retained participation dimensions. People with and without aphasia showed similar participation retention for physically demanding activities of between 45% and 49%; and both groups retained participation in communicatively- and cognitively-demanding activities at higher rates of about 60–70%. Having aphasia, at least in this sample of post-stroke individuals, does not constitute a greater barrier to participation in several different types of activities than having a stroke alone, using the participation measures we derived for this study. Of course, individuals with more severe post-stroke deficits, both in the motor or language domains, may experience greater restrictions in participation. Although participation rates are reduced in this “milder” stroke sample, the fact that 60–70% of high-communicative or high-cognitive activities are retained may also reflect the value that people post-stroke place on these particular activities. For example, 15 of the 35 high-communicative activities are within the ACS domain of “social activities,” and as such, these activities are likely important to quality of life post-stroke.

The regression analyses that examined whether self-perception of impairments in the three domains predicted participation post-stroke also revealed some interesting and unexpected findings. Self-perception of physical functioning and chronological age emerged as predictors of participation in physically demanding activities as expected. The fact that age was a predictor is not unexpected given known changes in physical functioning related to age even in healthy aging, let alone post-stroke. But self-perception of physical functioning also uniquely predicted participation in cognitively demanding activities, which was not expected. A weaker relationship was also seen between self-perception of physical function and participation in communicatively-demanding activities in the original regression without covarying for age, education, gender, and NIHSS score.

Why should self-perception of physical functioning emerge as the prominent predictor for activity retention of high-cognitive or high-communicative activities? Some of the effect may be explained by the fact that some activities are high on two or more of the dimensions. For example, of the 23 high-cognitive activities, 8 are also high-physical; of the 35 high-communicative, 4 are also high physical. When people are rating themselves on the Stroke Impact Scale are they perceiving their physical impairments as more important than impairments in other areas? Are they poor judges of their non-physical selves? Or do they just know something is wrong and cannot attribute it to a specific cause?

Considering the lack of relationship obtained between self-perception of communication and cognitive skills and retention of communicatively- or cognitively-demanding activities, we can ask the same question: Why do self-perceptions of communicative or cognitive challenges appear *not* to predict retention of communicatively- or cognitively-demanding activities? There are several possibilities. One is that self-perception ratings, particularly in these non-physical domains, may not be reliable, in the sense that they may not coincide with actual performance measures. Tucker et al. (44) found a systematicity in self-report for various measures of self-perception ratings within domains like social and physical functioning, such that people were consistent in how they perceived themselves across measures, even if the self- perception scores did not coincide with reality on more objective measures.

Furthermore, individuals post-stroke have varied personal reactions to changes in functioning. For example, some individuals with mild aphasia may perceive their impairment as severe, whereas other individuals with moderate to severe aphasia do not perceive their communication impairment as debilitating. Some people with right hemisphere damage may frankly not perceive any communication or cognitive impairment when compared to outside observers and on objective measurements their communication and cognition fall in the disordered range. When using patient-reported measures such as those in this study, there may be an impression of unreliability to outside observers; nevertheless this may reflect the patient's true perception of their reality.

Another possibility is that people are resourceful post-stroke and have developed numerous compensations for their communication and cognitive challenges such that despite aphasia and other cognitive impairments they find ways to express themselves and participate in valued life activities, though not to the degree they did before, thus reporting that their impairments are few. In contrast, they may perceive (rightly or wrongly) that there are fewer ways to easily compensate for physical impairments, thereby diminishing the relationship between perceptions of physical function and reported participation in physically-demanding activities. In fact, some activities requiring high physical exertion may simply not be able to be easily adapted and thus result in lower retention rates.

Future study should also consider how best to interpret patient-centered or self-perception measures. Could a valid measure of self-awareness inform how self-perception scores are interpreted by assisting in sorting out people with poor self-awareness from those with good self-awareness? Self-perception ratings serve an important purpose in insuring that targets of interventions are important to individuals and allow us to measure outcomes of importance to people post-stroke. But they may not be as useful for people who are poor judges of their own abilities. In a subsequent investigation we aim to conduct a similar study but use objective measures, rather than self-perception measures of functioning in these three domains (communication, cognition, and physical function). Comparisons of self-perception scores to objective measures may highlight different groups of individuals in which these scores are congruent or not. It is also possible that objective measures would show a more direct relationship to retention of participation in the three domains covered in this study than the self-perception measures.

Beyond the limitations discussed above with respect to self-perception measures, other limitations of this study include the fact that the sample was somewhat limited in range of stroke severity and that we had more individuals who were women than men. It is also possible that the group of people with aphasia had milder aphasia, resulting in lack of group differences between those with and without aphasia. Although we accommodated the communication deficit in the group of people with aphasia using the strategies outlined in Tucker et al. (44) it is also possible that in some cases these accommodations were not sufficient to render fully reliable results on the self-perception measures used in this study. Another limitation in exploring factors related to participation is that we only looked at three dimensions (communication, cognition, and physical exertion). Some of the other dimensions that ACS items may be rated high on (for example needing a partner to do the activity with, financial resources, or need for transportation) could have been more important to activity retention rates than the factors that were considered [see Foley et al. (3)]. Future research will need to more fully address the variety of personal as well as environmental factors that ultimately affect participation in life activities post-stroke.

Moreover, this study is limited in that a modest proportion of variance was accounted for by the examined regression models. For the six models tested (three participation outcomes × with/without covariates) the *R*^2^ values ranged from 0.22 to 0.34. Clearly, there is a large proportion of the total variance in participation unaccounted for in this investigation. Future work will be needed to test other possible predictors of participation and more complex relationships among the predictors themselves to account for variance in participation.

Further, because this study used the ACS as its primary outcome measure there are limits to the interpretation of the results of this study. The ACS focuses on the retention of pre-stroke activities. Although there were no differences in the retention of pre-stroke activities, we do not know the perceived quality of participation based on the ACS. It is quite possible that PWA are more dissatisfied or feel some restrictions in their capabilities to engage in activities after stroke. Even though they continue to participate, PWA may feel that their language impairment reduces their satisfaction with participation or may alter their engagement or enjoyment of those activities. Futher work, perhaps including a qualitative study, is warranted to understand the nature of activity participation after stroke and potential predictors of participation satisfaction.

Rehabilitation professionals may want to consider the results of this study in their clinical practice. First, even those with mild stroke will experience significant restrictions in their pre-stroke activities, even after rehabilitation—on the order of 30–50%. In addition, self-perception of physical function is the only self-perception rating that relates to participation retention in cognitively-demanding activities and physically-demanding activities. No self-perception rating that we examined related to participation in communicatively-demanding activities. Importantly, a significant proportion of the variance in participation scores was unaccounted for by self-perception and stroke severity ratings. Therefore, there is still much to learn about facilitators and inhibitors of post-stroke activity participation.

## Data Availability Statement

The datasets generated for this study are available on request to the corresponding author.

## Ethics Statement

The studies involving human participants were reviewed and approved by Washington University Human Research Protection Office and Partners HealthCare Institutional Review Board. The patients/participants provided their written informed consent to participate in this study.

## Author Contributions

LC and MN contributed to the conception, design of the work, interpretation of the findings, and drafted the manuscript. KB and JM acquired the data. AF designed the statistical approach and conducted the analyses. KB, JM, and CB revised it critically for important intellectual content. All authors contributed to manuscript revision, read, and approved the submitted version.

## Conflict of Interest

The authors declare that the research was conducted in the absence of any commercial or financial relationships that could be construed as a potential conflict of interest.
