# Supplementary material for: Self-Perception of Physical Function Contributes to Participation in Cognitively- and Physically-Demanding Activities After Stroke
Source: Front Neurol. 2020 Jun 9;11:474. doi: 10.3389/fneur.2020.00474 (PMC7296112; doi:10.3389/fneur.2020.00474)
Supplement: Supplementary file 1 [file Appendix.pdf]

Appendix I. The 59 items of the ACS that were high on one or more of the three dimensions (communication, cognition, and physical exertion), in ACS item order. Shaded cells show high scores at or above 2.0. Average percent retained scores for each item for the entire group of participants are in the right-most column.

ACS Item	ACS Subcategory	Cognitive	Commun-ication	Physical Exertion	Average % retained for entire group
**01.**Shopping in a Store	Instrumental ADL	2.2	2.2	1.6	74
**02.**Shopping for Groceries	Instrumental ADL	2.2	2.3	1.6	83
**05.**Yard Maintenance	Instrumental ADL	1.5	0.3	2.6	47
**07.** Cooking Dinner	Instrumental ADL	2.2	1.2	1.4	80
**08.**Household Maintenance	Instrumental ADL	2.1	0.8	2.1	47
**09.**Fixing Things Around the House	Instrumental ADL	2.3	1.0	2.0	45
**10.**Driving	Instrumental ADL	2.8	2.1	1.2	61
**12.**Car Maintenance	Instrumental ADL	2.7	1.2	2.0	46
**13.**Going to Doctor or Therapy	Instrumental ADL	1.7	2.5	1.4	96
**15.**Paying Bills	Instrumental ADL	2.8	2.7	0.5	80
**16.**Managing Investment	Instrumental ADL	2.9	2.6	0.4	79
**19.**Child Care	Instrumental ADL	2.6	2.4	2.5	58
**20.**Work (paid)		2.8	2.9	2.3	26

	Instrumental ADL				
**28.**Computer (email, paying bills, shopping)	Low Demand Leisure	2.4	2.7	0.6	77
**31.**Playing Cards (solitaire, poker, bridge)	Low Demand Leisure	2.2	1.5	0.6	68
**32.**Putting together Puzzles	Low Demand Leisure	2.1	0.5	0.6	67
**33.**Crossword or Sudoku Puzzle	Low Demand Leisure	2.5	1.9	0.4	77
**37.**Playing a Musical Instrument	Low Demand Leisure	2.2	1.4	1.6	41
**38.**Reading Magazines/Books	Low Demand Leisure	2.3	2.8	0.4	82
**39.**Reading Newspaper	Low Demand Leisure	2.3	2.8	0.4	82
**40.**Reading the Bible/Religious Materials	Low Demand Leisure	2.3	2.8	0.4	86
**41.**Singing in Choir or Group	Low Demand Leisure	1.5	2.1	1.4	67
**42.**Creative Writing/Journal	Low Demand Leisure	2.1	2.3	0.5	63
**43.**Letter Writing	Low Demand Leisure	2.1	2.5	0.6	57
**50.**Going to the Theater	Low Demand Leisure	1.0	2.0	0.9	71
**51.**Watching Movies	Low Demand Leisure	0.8	2.4	0.2	93
**52.**Watching Television	Low Demand Leisure	0.8	2.5	0.2	98
**53.**Listening to Music	Low Demand Leisure	0.5	2.0	0.1	93
**54.**Listening to Radio	Low Demand Leisure	0.6	2.1	0.1	88
**56.**Swimming	High Demand Leisure	1.4	0.3	2.8	43
**57.**Playing Team Sports	High Demand Leisure	2.3	2.1	3.0	24
**58.**Woodworking	High Demand Leisure	2.1	0.6	2.2	30
**59.**Bowling	High Demand Leisure	1.6	1.0	2.2	34

**60.**Golfing	High Demand Leisure	1.8	0.9	2.4	33
**61.**Walking	High Demand Leisure	0.7	0.2	2.1	76
**62.**Running	High Demand Leisure	0.9	0.2	2.9	33
**63.**Exercising	High Demand Leisure	1.1	0.5	2.8	75
**64.**Yoga/Pilates/Tai Chi	High Demand Leisure	1.5	0.9	2.6	64
**65.**Playing Tennis or other Racquet Sports	High Demand Leisure	1.8	1.1	2.9	24
**66.** Hiking	High Demand Leisure	1.1	0.4	2.9	46
**67.**Bicycling	High Demand Leisure	1.2	0.4	2.9	27
**68.**Yard Games	High Demand Leisure	1.5	1.1	2.2	54
**69.**Camping	High Demand Leisure	1.9	0.9	2.3	44
**70.**Canoeing/Boating/Sailing	High Demand Leisure	1.9	0.9	2.6	38
**73.**Studying for Personal Advancement	Social	2.9	2.9	0.5	57
**74.**Traveling Local/Regional	Social	2.3	2.3	1.9	65
**75.**Traveling National/International	Social	2.4	2.3	2.1	68
**76.**Parties/Picnics	Social	1.3	2.2	1.5	74
**77.**Family Gatherings	Social	1.2	2.3	1.4	84
**78.**Talking on the Telephone	Social	1.5	2.7	0.3	89
**79.**Visiting with Family/Friends who are Ill	Social	1.3	2.3	1.0	77
**80.**Visiting with Friends	Social	1.3	2.4	1.0	78
**81.**Eating at a Restaurant	Social	1.3	2.0	0.9	93
**82.**Dancing	Social	1.0	0.7	2.7	46

**84.** Volunteer Work	Social	1.8	2.0	1.9	47
**86.** Storytelling with Children	Social	2.0	2.6	0.6	63
**87.** Being with a Spouse or Partner	Social	1.5	2.1	0.9	82
**88.** Dating/Spending Time with Friends	Social	1.7	2.4	1.2	77
**89.** Entertaining at Home or Club	Social	1.9	2.3	1.6	74

Appendix II. All items from the Stroke Impact Scale (version 2.0) used as predictor measures. All items were rated on a scale of 1-5, with 5 representing good function.

Strength

In the past week, how would you rate the strength in your. . .

a. Arm that was <u>most affected</u> by your stroke?
b. Grip of your hand that was <u>most affected</u> by your stroke?
c. Leg that was <u>most affected</u> by your stroke?
d. Foot/ankle that was <u>most affected</u> by your stroke?

Hand Function

In the past 2 weeks, how difficult was it to use your hand that was most affected by your stroke to. . .

a. Carry heavy objects (e.g., bag of groceries)?
b. Turn a doorknob?
c. Open a can or jar?
d. Tie a shoe lace?
e. Pick up a dime?

Mobility

In the past 2 weeks, how difficult was it to . . .

a. Stay sitting without losing your balance?
b. Stay standing without losing your balance?
c. Walk without losing your balance?
d. Move from a bed to a chair?
e. Get out of a chair without using your hands for support?
f. Walk one block?
g. Walk fast?
h. Climb one flight of stairs?
i. Climb several flights of stairs?
j. Get in and out of a car?

Communication

In the past week, how difficult was it to. . .

a. Say the name of someone who was in front of you?
b. Understand what was being said to you in a conversation?
c. Reply to questions?
d. Correctly name objects?
e. Participate in a conversation with a group of people?
f. Have a conversation on the telephone?
g. Call another person on the telephone, including selecting the correct phone number and dialing?

Memory and Thinking

In the past week, how difficult was it for you to. . .

a. Remember things that people just told you?
b. Remember things that happened the day before?
c. Remember to do things (e.g., keep schedule appointments or take medication)?
d. Remember the day of the week?
e. Add and subtract numbers?
f. Concentrate?
g. Think quickly?
h. Solve everyday problems?
